# Harlestonensis, in Answer to W. G.

**Published:** 1808-06

**Authors:** 


### *To the Editors of the Medical and Physical Journal.*

GENTLEMEN,

IT did not occur to me, when I was writing the paper which was inserted in the last Number of your Journal, that the language employed, or any thing there stated, could at all call forth the angry feelings, or excite disagreeeable sensations in the minds of any of your readers. But, it seems, I was mistaken; for your Correspondent, who signs himself W. G. M. D. has taken it in dudgeon, that I should presume to question the propriety of the administration of mercury in typhus; because, forsooth, it turns out that he himself thinks highly of it. I was not aware that I was treading on tender or consecrated ground, or that the good Doctor was so irritable or sore on the subject of his favorite remedy, which, he tells us, has been so very successful in *his* hands; successful, indeed, it must have been, when he says he has used it for these twenty years with *constant* relief to his patients, and, I dare say, with equal benefit to himself.

I know not why W. G. should take up this subject so hastily, as, most assuredly, there was no intention of making any *attack* or observation either on him or his practice in particular. The paper with the signature *Harlestonensis*, was sent in answer to one by Dr. Mease; and, I trust, the observagangrene, into which both kinds of inflammation is liable to go, will require some modified plan of treatment, according to the nature of the previous inflammation.

observations it contained were made without either *arrogance* or *petulance*; and I now call upon W. G. to stand forth, and point out the passage he would construe as *sarcastic* or *invidious*. Opinions given to the public are open to criticism, and if that criticism be conducted with candour and liberality, and do not go beyond just limits, I know of no reason why any man should take offence, or arrogate to himself an importance which does not belong to him. For myself I can say, I shall always feel at liberty to make my remarks, without the fear of calling down the resentment of your Yarmouth Correspondent; and he may rest assured that (though he has M. D. tacked to his tail) the *precaution*, as he is pleased to call it, in not affixing my real name, did not arise from any dread of his doctorial consequence, or his *correct philosophical arguments*. It is the misfortune of some men to render themselves conspicuously ridiculous upon every occasion which offers; to such men occasions are seldom wanting; and the Doctor seems to have been determined not to let the opportunity slip of verifying the observation in his own person.

Notwithstanding the opinion he entertains of his own *penetration* in discovering the writer of the paper in question, and the little trouble it cost him to find out the Author, of what indeed one would be inclined to think the Doctor considers as treason against mercury, yet he is so completely in the dark as to the person of the writer, that really he is entitled to all the pity that can be bestowed upon him for his *want of penetration*, and to contempt for his conduct, which is more personal than philosophical. The fact is, that I know but little of either his character or his person, but by report; and, if I am rightly informed, he is not a little bigoted to mercury, but is entitled to that mercurial celebrity of which he appears so jealous. He has yet to learn I am not that juvenile practitioner he supposes; I have been in practice some years, and therefore it is presumed I may be allowed to judge for myself, without being subjected to his outrageous indecency, or his insinuations. I do not know that I ever saw W. G. and therefore he will not be believed when he says, that *I have seen many cases with him; that he always treated me in a gentleman-like manner, both in and out of the profession; and has been a useful and sincere friend.* And this he says, without *exaggeration*. Now, it is a little unfortunate for him, that his assertions are couched in such positive terms, as it becomes necessary for me to state, that not one word he he has written respecting me is true, but positively false and this *I say without exaggeration.*

One piece of advice I should recommend to this Correspondent of yours; which is, that he does not, in future, suffer himself to be led away by the impulse of the moment, but to take more time for consideration, that he may not render himself ridiculous in the eyes of every one; and perhaps it would be better for him if he did not hug himself in the conceit that he was meant, when, in all probability, he was too insignificant to have been thought of by

HARLESTONENSIS.

*May* 14, 1808.

